# 2-Amino-6-methyl-4,5,6,7-tetra­hydro-1-benzothio­phene-3-carbonitrile

**DOI:** 10.1107/S1600536811006076

**Published:** 2011-02-23

**Authors:** Mohamed Ziaulla, Afshan Banu, Noor Shahina Begum, Shridhar I. Panchamukhi, I. M. Khazi

**Affiliations:** a Department of Studies in Chemistry, Bangalore University, Bangalore 560 001, India; b Department of Chemistry, Karnatak University, Dharwad 580 003, India

## Abstract

In the title compound, C_10_H_12_N_2_S, one of the C atoms of the cyclo­hexene ring (at position 6) and the methyl group attached to it are disordered over two sets of sites in a 0.650 (3):0.350 (3) ratio. The cyclo­hexene ring in both the major and minor occupancy conformers adopts a half-chair conformation. The thio­phene ring is essentially planar (r.m.s. deviation = 0.05 Å). In the crystal, N—H⋯N hydrogen bonds involving the amino groups result in inversion dimers with *R*_2_^2^(12) graph-set motif. Further N—H⋯N hydrogen bonds involving the amino and carbonitrile groups generate zigzag chains along the *a* axis.

## Related literature

For preparation of the title compound, see: Shetty *et al.* (2009 ▶). For general background to benzothio­phenes, see: Katritzky *et al.* (1996 ▶); Shishoo & Jain (1992 ▶). For related structures, see: Akkurt *et al.* (2008 ▶); Harrison *et al.* (2006 ▶); Vasu *et al.* (2004 ▶). For graph-set notation, see: Bernstein *et al.* (1995 ▶).
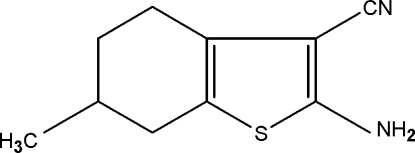


## Experimental

### 

#### Crystal data

C_10_H_12_N_2_S
*M* *_r_* = 192.29
Monoclinic, 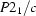

*a* = 9.0415 (2) Å
*b* = 8.3294 (2) Å
*c* = 13.1283 (3) Å
β = 90.169 (2)°
*V* = 988.69 (4) Å^3^
*Z* = 4
Mo *K*α radiation
μ = 0.28 mm^−1^
*T* = 123 K
0.16 × 0.16 × 0.14 mm

#### Data collection

Bruker SMART APEX CCD detector diffractometer
Absorption correction: multi-scan (*SADABS*; Bruker, 1998 ▶) *T*_min_ = 0.957, *T*_max_ = 0.962
11284 measured reflections
2441 independent reflections
1878 reflections with *I* > 2σ(*I*)
*R*_int_ = 0.039

#### Refinement

*R*[*F* ^2^ > 2σ(*F* ^2^)] = 0.040
*wR*(*F* ^2^) = 0.107
*S* = 1.07
2441 reflections
127 parameters
H-atom parameters constrained
Δρ_max_ = 0.39 e Å^−3^
Δρ_min_ = −0.30 e Å^−3^

### 

Data collection: *SMART* (Bruker, 1998 ▶); cell refinement: *SAINT-Plus* (Bruker, 1998 ▶); data reduction: *SAINT-Plus*; program(s) used to solve structure: *SHELXS97* (Sheldrick, 2008 ▶); program(s) used to refine structure: *SHELXL97* (Sheldrick, 2008 ▶); molecular graphics: *CAMERON* (Watkin *et al.*, 1996 ▶); software used to prepare material for publication: *WinGX* (Farrugia, 1999 ▶).

## Supplementary Material

Crystal structure: contains datablocks global, I. DOI: 10.1107/S1600536811006076/pv2384sup1.cif

Structure factors: contains datablocks I. DOI: 10.1107/S1600536811006076/pv2384Isup2.hkl

Additional supplementary materials:  crystallographic information; 3D view; checkCIF report

## Figures and Tables

**Table 1:** Hydrogen-bond geometry (Å, °)

*D*—H⋯*A*	*D*—H	H⋯*A*	*D*⋯*A*	*D*—H⋯*A*
N2—H2*A*⋯N1^i^	0.88	2.22	3.087 (2)	170
N2—H2*B*⋯N1^ii^	0.88	2.41	3.247 (2)	160

Symmetry codes: (i) 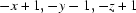; (ii) 
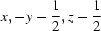
.

**Fig. 1.**
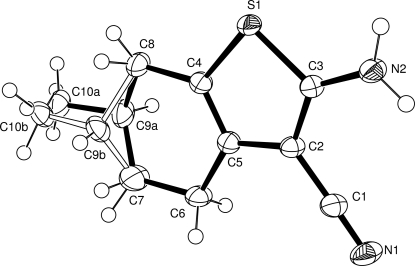
ORTEP (Farrugia, 1999) view of the title compound, showing 50% probability ellipsoids and the atom numbering scheme; C-atoms C9b and C10b represent the minor conformer.

**Fig. 2.**
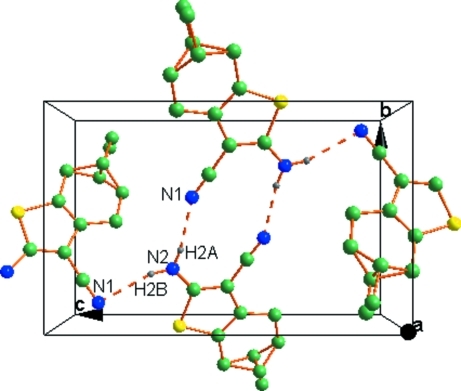
A unit cell packing of the title compound showing intermolecular interactions with dotted lines. H-atoms not involved in hydrogen bonding have been excluded.

## supplementary crystallographic information

### Comment

Benzothiophenes are important heterocycles either as biological active molecules or as luminescent components used in organic materials (Shishoo & Jain, 1992; Katritzky *et al.*, 1996). In this paper, we report the crystal structure of a benzothiophene derivative.

In the title compound (Fig. 1), the fused benzothiophene ring system is substituted with amino, methyl and carbonitrile groups. The carbon atoms C9 and C10 are disordered over two sites (C9A/C9B and C10A/C10B) with site occupancy factors 0.650 (3) and 0.350 (3) resulting in a major and a minor conformers. The cyclohexene ring in both conformers is in a half-chair conformation with C9A and C9B 0.547 (4) and 0.506 (6) Å, respectively, displaced on the opposite sides from the plane formed by the rest of the ring C-atoms (max. deviation being 0.063 (2) Å for C6). The thiophene ring is essentially planar. In several benzothiophene derivatives the cyclohexyl ring adopts half-chair conformation (Akkurt *et al.*, 2008; Harrison *et al.*, 2006; Vasu *et al.*, 2004). The crystal structure is stabilized by two types of N—H···N intermolecular interactions (Table 1); N2—H2A···N1 hydrogen bond forms centrosymmetric, head-to-head dimers about inversion centers corresponding to graph set *R*^2^_2_(12) motif(Bernstein *et al.*, 1995) while N2—H2B···N1 hydrogen bonds generate chains of molecules in a zigzag pattern along the *a* axis (Fig. 2).

### Experimental

The title compound was synthesized by following the procedure reported earlier (Shetty *et al.*, 2009).

### Refinement

The H atoms were placed at calculated positions in the riding model approximation with N—H = 0.88 Å and C—H = 0.98, 0.99 and 1.00 Å for methylene, methyl and methyne type H-atoms, respectively; *U*_iso_(H) = 1.2*U*_eq_(N/non-methyl C) and 1.5*U*_eq_(methyl C).

### Crystal data

**Table:**

C_10_H_12_N_2_S	*F*(000) = 408
*M**_r_* = 192.29	*D*_x_ = 1.292 Mg m^−^^3^
Monoclinic, *P*2_1_/*c*	Mo *K*α radiation, λ = 0.71073 Å
Hall symbol: -P 2ybc	Cell parameters from 2441 reflections
*a* = 9.0415 (2) Å	θ = 2.9–29.2°
*b* = 8.3294 (2) Å	µ = 0.28 mm^−^^1^
*c* = 13.1283 (3) Å	*T* = 123 K
β = 90.169 (2)°	Block, yellow
*V* = 988.69 (4) Å^3^	0.16 × 0.16 × 0.14 mm
*Z* = 4	

### Data collection

**Table:**

Bruker SMART APEX CCD detector diffractometer	2441 independent reflections
Radiation source: Enhance (Mo) X-ray Source	1878 reflections with *I* > 2σ(*I*)
graphite	*R*_int_ = 0.039
ω scans	θ_max_ = 29.2°, θ_min_ = 2.9°
Absorption correction: multi-scan (*SADABS*; Bruker, 1998)	*h* = −11→12
*T*_min_ = 0.957, *T*_max_ = 0.962	*k* = −11→10
11284 measured reflections	*l* = −17→16

### Refinement

**Table:**

Refinement on *F*^2^	Primary atom site location: structure-invariant direct methods
Least-squares matrix: full	Secondary atom site location: difference Fourier map
*R*[*F*^2^ > 2σ(*F*^2^)] = 0.040	Hydrogen site location: inferred from neighbouring sites
*wR*(*F*^2^) = 0.107	H-atom parameters constrained
*S* = 1.07	*w* = 1/[σ^2^(*F*_o_^2^) + (0.0536*P*)^2^ + 0.2028*P*] where *P* = (*F*_o_^2^ + 2*F*_c_^2^)/3
2441 reflections	(Δ/σ)_max_ = 0.001
127 parameters	Δρ_max_ = 0.39 e Å^−^^3^
0 restraints	Δρ_min_ = −0.30 e Å^−^^3^

### Special details

**Table:**

Experimental. The compound was synthesized by following the procedure given in NitinKumar *et al.*, (2009)
Geometry. All s.u.'s (except the s.u. in the dihedral angle between two l.s. planes) are estimated using the full covariance matrix. The cell s.u.'s are taken into account individually in the estimation of s.u.'s in distances, angles and torsion angles; correlations between s.u.'s in cell parameters are only used when they are defined by crystal symmetry. An approximate (isotropic) treatment of cell s.u.'s is used for estimating s.u.'s involving l.s. planes.
Refinement. Refinement of *F*^2^ against ALL reflections. The weighted *R*-factor *wR* and goodness of fit *S* are based on *F*^2^, conventional *R*-factors *R* are based on *F*, with *F* set to zero for negative *F*^2^. The threshold expression of *F*^2^ > 2σ(*F*^2^) is used only for calculating *R*-factors(gt) *etc*. and is not relevant to the choice of reflections for refinement. *R*-factors based on *F*^2^ are statistically about twice as large as those based on *F*, and *R*- factors based on ALL data will be even larger.

### Fractional atomic coordinates and isotropic or equivalent isotropic displacement parameters (Å^2^)

**Table:**

	*x*	*y*	*z*	*U*_iso_*/*U*_eq_	Occ. (<1)
S1	0.68698 (5)	0.02703 (5)	0.34885 (3)	0.02519 (15)	
N1	0.5603 (2)	−0.40456 (18)	0.60740 (11)	0.0378 (4)	
N2	0.54766 (17)	−0.25706 (17)	0.33230 (10)	0.0292 (4)	
H2A	0.5129	−0.3480	0.3565	0.035*	
H2B	0.5362	−0.2336	0.2674	0.035*	
C1	0.5991 (2)	−0.3000 (2)	0.55684 (12)	0.0263 (4)	
C2	0.64719 (19)	−0.16794 (19)	0.49726 (12)	0.0224 (4)	
C3	0.61829 (18)	−0.15372 (19)	0.39455 (12)	0.0217 (3)	
C4	0.75534 (19)	0.0810 (2)	0.46849 (12)	0.0251 (4)	
C5	0.72696 (19)	−0.0328 (2)	0.53858 (12)	0.0236 (4)	
C6	0.7762 (2)	−0.0180 (2)	0.64700 (13)	0.0303 (4)	
H6A	0.8312	−0.1158	0.6670	0.036*	
H6B	0.6885	−0.0088	0.6916	0.036*	
C7	0.8734 (3)	0.1266 (3)	0.66129 (16)	0.0514 (6)	
H7A	0.8688	0.1572	0.7341	0.062*	0.650 (3)
H7B	0.9764	0.0931	0.6474	0.062*	0.650 (3)
C8	0.8387 (2)	0.2343 (2)	0.48637 (13)	0.0328 (4)	
H8A	0.7892	0.3236	0.4500	0.039*	0.650 (3)
H8B	0.9405	0.2241	0.4596	0.039*	0.650 (3)
C9A	0.8440 (3)	0.2705 (3)	0.6012 (2)	0.0296 (5)	0.650 (3)
H9AA	0.7434	0.3094	0.6209	0.036*	0.650 (3)
C10A	0.9518 (5)	0.4053 (5)	0.6248 (3)	0.0429 (10)	0.650 (3)
H10A	0.9544	0.4241	0.6985	0.064*	0.650 (3)
H10B	0.9199	0.5034	0.5900	0.064*	0.650 (3)
H10C	1.0508	0.3753	0.6013	0.064*	0.650 (3)
H7C	0.8128	0.2072	0.6977	0.062*	0.350 (3)
H7D	0.9527	0.0937	0.7091	0.062*	0.350 (3)
H8C	0.7690	0.3238	0.4988	0.039*	0.350 (3)
H8D	0.8997	0.2611	0.4262	0.039*	0.350 (3)
C9B	0.9414 (6)	0.2065 (6)	0.5837 (4)	0.0296 (5)	0.350 (3)
H9BA	1.0221	0.1334	0.5601	0.036*	0.350 (3)
C10B	1.0183 (9)	0.3644 (11)	0.6111 (7)	0.0429 (10)	0.350 (3)
H10D	0.9449	0.4508	0.6140	0.064*	0.350 (3)
H10E	1.0924	0.3897	0.5592	0.064*	0.350 (3)
H10F	1.0669	0.3537	0.6775	0.064*	0.350 (3)

### Atomic displacement parameters (Å^2^)

**Table:**

	*U*^11^	*U*^22^	*U*^33^	*U*^12^	*U*^13^	*U*^23^
S1	0.0327 (3)	0.0257 (2)	0.0171 (2)	−0.00706 (18)	−0.00294 (16)	0.00388 (16)
N1	0.0684 (12)	0.0241 (8)	0.0207 (8)	−0.0089 (8)	−0.0066 (7)	0.0026 (6)
N2	0.0453 (9)	0.0247 (8)	0.0175 (7)	−0.0093 (7)	−0.0035 (6)	0.0003 (6)
C1	0.0395 (10)	0.0207 (8)	0.0187 (8)	−0.0005 (7)	−0.0035 (7)	−0.0027 (7)
C2	0.0287 (9)	0.0203 (8)	0.0183 (8)	0.0000 (7)	−0.0001 (6)	0.0010 (6)
C3	0.0241 (8)	0.0207 (8)	0.0203 (8)	−0.0003 (6)	0.0011 (6)	0.0004 (6)
C4	0.0296 (9)	0.0270 (9)	0.0185 (8)	−0.0053 (7)	−0.0033 (7)	0.0010 (7)
C5	0.0263 (9)	0.0243 (8)	0.0202 (8)	−0.0017 (7)	−0.0011 (7)	0.0004 (7)
C6	0.0427 (11)	0.0294 (9)	0.0189 (9)	−0.0041 (8)	−0.0057 (7)	0.0030 (7)
C7	0.0744 (17)	0.0471 (13)	0.0327 (11)	−0.0244 (12)	−0.0248 (11)	0.0067 (9)
C8	0.0418 (11)	0.0330 (10)	0.0237 (9)	−0.0159 (8)	−0.0040 (8)	0.0028 (7)
C9A	0.0321 (14)	0.0302 (13)	0.0265 (12)	−0.0074 (10)	−0.0027 (11)	−0.0028 (10)
C10A	0.047 (3)	0.052 (2)	0.0294 (16)	−0.029 (2)	−0.0007 (19)	−0.0052 (15)
C7A	0.0744 (17)	0.0471 (13)	0.0327 (11)	−0.0244 (12)	−0.0248 (11)	0.0067 (9)
C8A	0.0418 (11)	0.0330 (10)	0.0237 (9)	−0.0159 (8)	−0.0040 (8)	0.0028 (7)
C9B	0.0321 (14)	0.0302 (13)	0.0265 (12)	−0.0074 (10)	−0.0027 (11)	−0.0028 (10)
C10B	0.047 (3)	0.052 (2)	0.0294 (16)	−0.029 (2)	−0.0007 (19)	−0.0052 (15)

### Geometric parameters (Å, °)

**Table:**

S1—C3	1.7363 (16)	C7—H7A	0.9900
S1—C4	1.7451 (17)	C7—H7B	0.9900
N1—C1	1.150 (2)	C8—C9A	1.538 (3)
N2—C3	1.347 (2)	C8—H8A	0.9900
N2—H2A	0.8800	C8—H8B	0.9900
N2—H2B	0.8800	C9A—C10A	1.519 (5)
C1—C2	1.419 (2)	C9A—H9AA	1.0000
C2—C3	1.378 (2)	C10A—H10A	0.9800
C2—C5	1.442 (2)	C10A—H10B	0.9800
C4—C5	1.346 (2)	C10A—H10C	0.9800
C4—C8	1.501 (2)	C9B—C10B	1.530 (10)
C5—C6	1.495 (2)	C9B—H9BA	1.0000
C6—C7	1.502 (3)	C10B—H10D	0.9800
C6—H6A	0.9900	C10B—H10E	0.9800
C6—H6B	0.9900	C10B—H10F	0.9800
C7—C9A	1.459 (3)		
			
C3—S1—C4	92.20 (8)	C9A—C7—H7A	107.6
C3—N2—H2A	120.0	C6—C7—H7A	107.6
C3—N2—H2B	120.0	C9A—C7—H7B	107.6
H2A—N2—H2B	120.0	C6—C7—H7B	107.6
N1—C1—C2	178.19 (17)	H7A—C7—H7B	107.0
C3—C2—C1	123.28 (15)	C4—C8—C9A	109.52 (16)
C3—C2—C5	113.22 (14)	C4—C8—H8A	109.8
C1—C2—C5	123.47 (14)	C9A—C8—H8A	109.8
N2—C3—C2	128.84 (15)	C4—C8—H8B	109.8
N2—C3—S1	120.93 (12)	C9A—C8—H8B	109.8
C2—C3—S1	110.22 (12)	H8A—C8—H8B	108.2
C5—C4—C8	126.07 (15)	C7—C9A—C10A	112.4 (3)
C5—C4—S1	111.48 (13)	C7—C9A—C8	112.0 (2)
C8—C4—S1	122.42 (12)	C10A—C9A—C8	111.3 (2)
C4—C5—C2	112.86 (15)	C7—C9A—H9AA	106.9
C4—C5—C6	122.40 (15)	C10A—C9A—H9AA	106.9
C2—C5—C6	124.73 (15)	C8—C9A—H9AA	106.9
C5—C6—C7	110.93 (15)	C10B—C9B—H9BA	105.4
C5—C6—H6A	109.5	C9B—C10B—H10D	109.5
C7—C6—H6A	109.5	C9B—C10B—H10E	109.5
C5—C6—H6B	109.5	H10D—C10B—H10E	109.5
C7—C6—H6B	109.5	C9B—C10B—H10F	109.5
H6A—C6—H6B	108.0	H10D—C10B—H10F	109.5
C9A—C7—C6	119.06 (19)	H10E—C10B—H10F	109.5
			
C1—C2—C3—N2	−2.3 (3)	C1—C2—C5—C4	−177.32 (17)
C5—C2—C3—N2	179.55 (17)	C3—C2—C5—C6	−178.44 (17)
C1—C2—C3—S1	177.36 (14)	C1—C2—C5—C6	3.4 (3)
C5—C2—C3—S1	−0.78 (19)	C4—C5—C6—C7	−6.7 (3)
C4—S1—C3—N2	−179.86 (15)	C2—C5—C6—C7	172.46 (19)
C4—S1—C3—C2	0.45 (13)	C5—C6—C7—C9A	34.4 (3)
C3—S1—C4—C5	0.01 (14)	C5—C4—C8—C9A	−18.3 (3)
C3—S1—C4—C8	178.33 (16)	S1—C4—C8—C9A	163.68 (16)
C8—C4—C5—C2	−178.70 (17)	C6—C7—C9A—C10A	179.9 (3)
S1—C4—C5—C2	−0.5 (2)	C6—C7—C9A—C8	−53.9 (3)
C8—C4—C5—C6	0.6 (3)	C4—C8—C9A—C7	42.0 (3)
S1—C4—C5—C6	178.83 (14)	C4—C8—C9A—C10A	168.8 (3)
C3—C2—C5—C4	0.8 (2)		

### Hydrogen-bond geometry (Å, °)

**Table:**

*D*—H···*A*	*D*—H	H···*A*	*D*···*A*	*D*—H···*A*
N2—H2A···N1^i^	0.88	2.22	3.087 (2)	170
N2—H2B···N1^ii^	0.88	2.41	3.247 (2)	160

Symmetry codes: (i) −*x*+1, −*y*−1, −*z*+1; (ii) *x*, −*y*−1/2, *z*−1/2.
